# Gastric antral vascular ectasia: A rare etiology of gastrointestinal bleeding in children

**DOI:** 10.1002/jpr3.70136

**Published:** 2025-12-22

**Authors:** Omar Alharbi, Sindhura Kasturi, George Yanni, Blake Rosenthal

**Affiliations:** ^1^ Division of Gastroenterology, Hepatology and Nutrition Children's Hospital Los Angeles (CHLA) Los Angeles California USA

**Keywords:** argon plasma coagulation, endoscopic therapy, hematemesis, melena, pediatric endoscopy

## Abstract

Gastrointestinal bleeding is relatively common in children. While most patients present with mild bleeding, gastric antral vascular ectasia (GAVE) is a rare but potentially life‐threatening cause. GAVE is typically associated with chronic conditions and more common in adults. The incidence, diagnosis, and management of GAVE in the pediatric population have not been established. We present two cases of GAVE with severe and chronic gastrointestinal bleeding. The first patient, a 7‐year‐old with chronic granulomatous disease who had melena and anemia following hematopoietic stem cell transplantation (HSCT). Endoscopic intervention with argon plasma coagulation (APC) was successful in achieving hemostasis. The second patient, an 18‐year‐old with veno‐occlusive disease (VOD), developed GAVE as a result of portal hypertension. Endoscopic intervention with APC successfully controlled the bleeding in this patient as well. These cases highlight the challenges associated with diagnosing and managing GAVE in children.

## 1 INTRODUCTION

Gastrointestinal (GI) bleeding is not an uncommon problem in children, with a reported incidence of 6%.^1^ Most cases of GI bleeding in children are mild, transient, and do not require any intervention. One of the rare but potentially life‐threatening causes of GI bleeding is gastric antral vascular ectasia (GAVE). More common in adults, GAVE can lead to acute or chronic GI bleeding that is often challenging to manage. The characteristic features of GAVE include small angiodysplastic lesions that appear as parallel vascular lines in the antrum of the stomach. Although the etiology of GAVE remains uncertain, it is known to be associated with several comorbid conditions, including chronic renal disease, liver cirrhosis, portal hypertension, and cardiovascular disorders.^2^, ^3^ GAVE has also been associated with hematopoietic stem cell transplantation (HSCT) in children.^4^ The incidence of GAVE in the pediatric population has not been established. However, in adults, it has been reported to comprise approximately 4% of all cases of nonvariceal GI bleeding.^5^ Here, we describe two pediatric cases of successful endoscopic management of GAVE and provide a review of the management of this rare condition.

## 2 CASE #1

**Figure 1:**
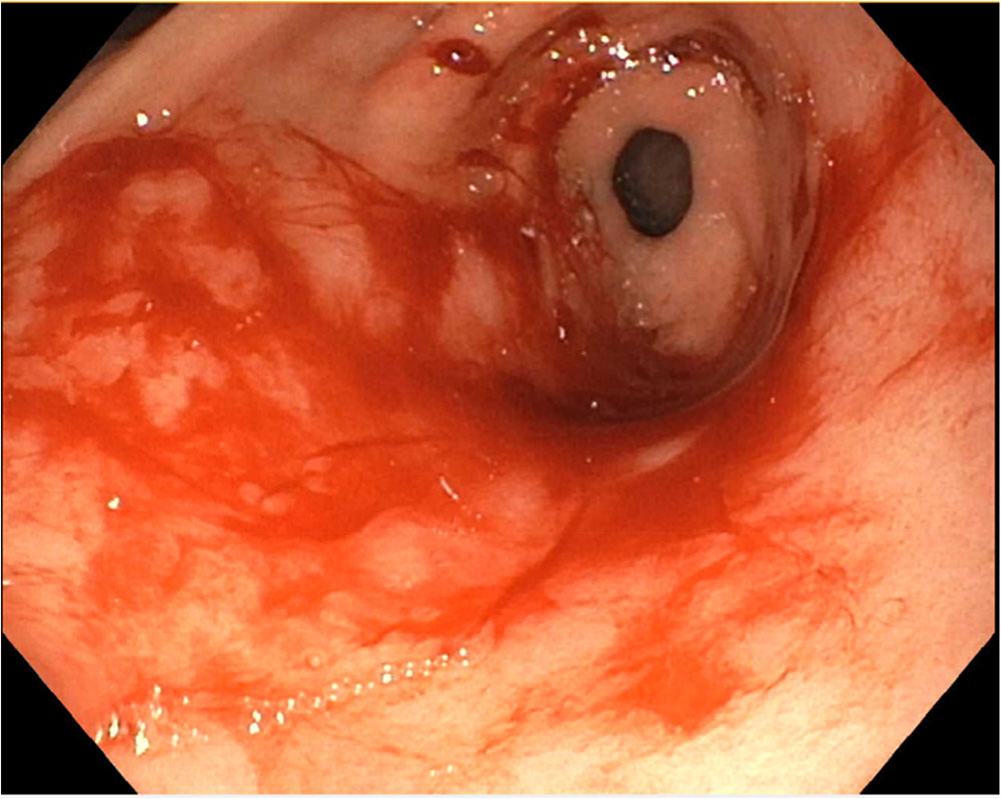
Patient #1 with active oozing in the antrum secondary to GAVE. GAVE, gastric antral vascular ectasia.

**Figure 2:**
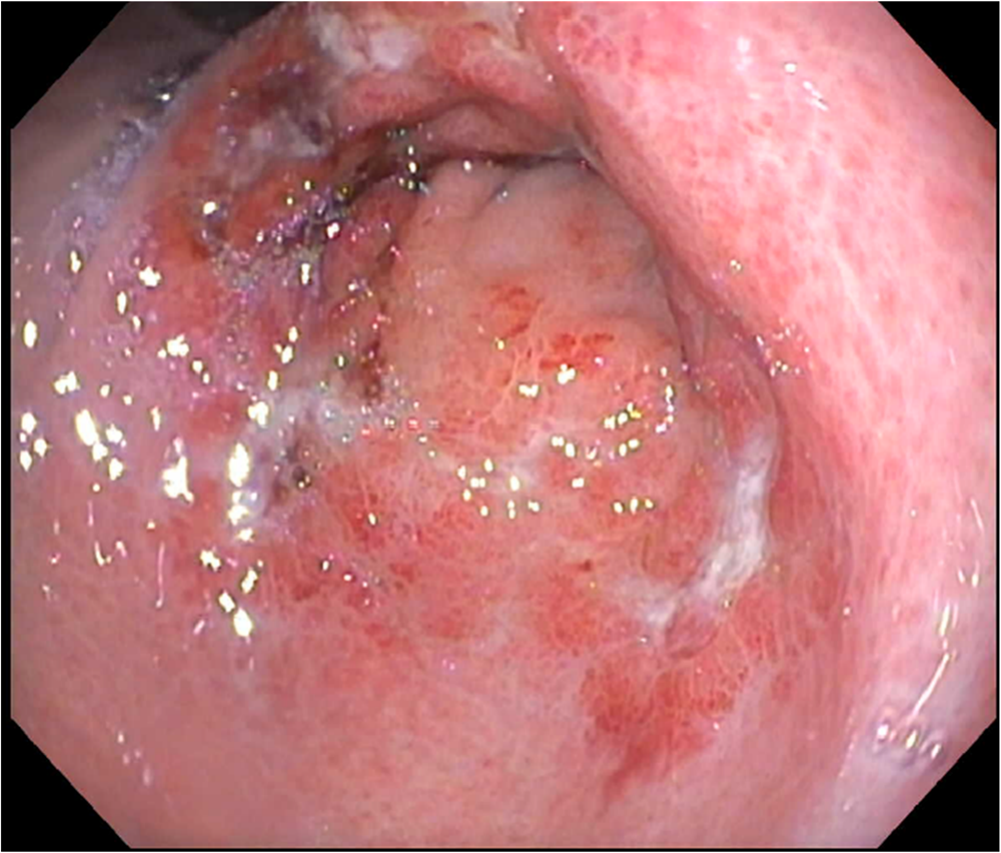
Patient #1 after multiple treatments with APC. APC, argon plasma coagulation.

Patient is a 7‐year‐old male with chronic granulomatous disease (CGD) status post matched unrelated donor (MUD) bone marrow transplant (BMT). Two months after his BMT, he developed melena and acute anemia requiring blood transfusion. His initial hemoglobin was 6.6 g/dL (10.7–13.4 g/dL). Computed tomography angiography (CTA) was performed, and it was negative for active bleeding or extravasation. He was started on proton pump inhibitor (PPI) and received packed red blood cells prior to endoscopic intervention. Upper esophagogastroduodenoscopy (EGD) was notable for a large amount of fresh blood upon entry into the stomach. After washing and suctioning the area, diffuse oozing of blood was noted throughout the antrum without ulceration or visible bleeding vessels (Figure 1). Diffuse bleeding would slowly return once washed away, suspicious for GAVE. Argon plasma coagulation (APC) was applied to the entire antral area, and hemostasis was achieved. We utilized the Erbe VIO 3 electrosurgical unit, using Forced APC setting (40–60 W). We used both types of APC catheters, straight and circumferential, to achieve adequate coagulation. Octreotide drip was initiated after endoscopy, and the patient remained on PPI. His octreotide drip was slowly weaned off after 5 days, and melena subsequently worsened with concomitant hemoglobin drop requiring transfusion. He underwent second endoscopy, which showed a large blood clot in the pylorus and active bleeding. Repeat APC treatment was performed extensively in the antrum, and hemostasis was achieved. After 5 days, melanotic stools recurred later requiring a third endoscopic intervention. Again, slow oozing of blood was noted in the antrum at the site of the previously treated area with APC. Repeat, extensive APC was applied in the antrum with minimal oozing noted at the end of the procedure. He underwent final endoscopic evaluation 7 days later, at which time no bleeding was noted in the antrum (Figure 2). His melena resolved, and hemoglobin stabilized without transfusion. The patient was discharged afterwards and has been following up in clinic with no evidence of recurrent GI bleeding for more than 15 months since his last endoscopy.

## 3 CASE #2

**Figure 3:**
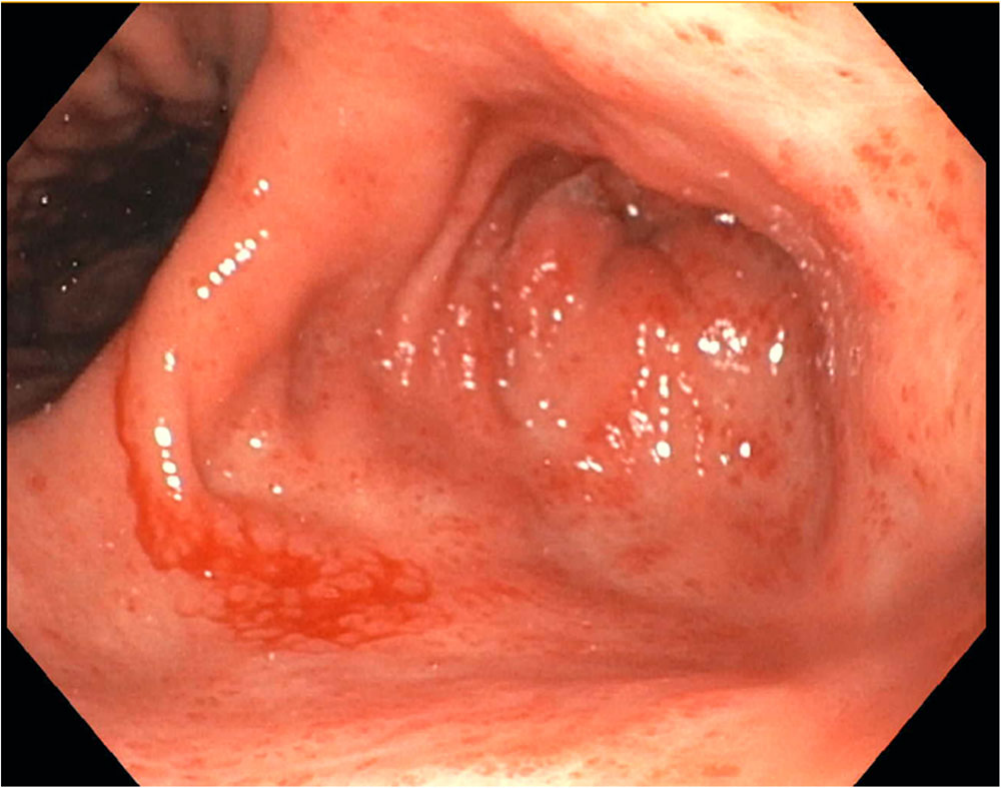
Patient #2 with active oozing in the antrum secondary to GAVE. GAVE, gastric antral vascular ectasia.

An 18‐year‐old female with a history of severe combined immunodeficiency has been under the care of the hepatology team at our institution for managing her veno‐occlusive disease (VOD). She developed portal hypertension with esophageal varices because of VOD. She underwent an urgent EGD for concerns of acute drop in hemoglobin noted during an outpatient visit. EGD revealed Grade 3 esophageal varices with red whale sign but with no active bleeding from these varices. In the stomach, diffuse portal hypertensive gastropathy (PHG) was observed, along with distinct findings in the antrum, including active bleeding that slowly reaccumulated from tortuous submucosal vessels (Figure 3). These findings in the antrum were suggestive of GAVE. The esophageal varices were banded, and APC was performed on the many small bleeding vessels in the antrum, achieving adequate hemostasis. We utilized the Erbe VIO 3 electrosurgical unit, using Forced APC setting (40–60 W) with straight and circumferential catheters. The patient underwent a second EGD after 2 weeks that showed improvement with no active bleeding in the antrum; however, small vascular lesions were still seen, for which APC was applied. EGD was repeated 2 months after the initial endoscopy. The antrum still had findings suggestive of GAVE but was overall improved without active bleeding or oozing. APC was reapplied to the area to enhance further healing. The patient had a follow‐up endoscopy 5 months later due to anemia, and there was no active bleeding in the treated area.

## 4 DISCUSSION

GAVE is an acquired condition of which the pathogenesis is not clearly understood to date. GAVE is typically localized to the antrum, but there are case reports of GAVE in different regions of the stomach, duodenum, jejunum, and rectum.^6^ Comorbid conditions are common in patients with GAVE, with 60% of GAVE patients having an autoimmune disorder and 30% of patients having concomitant liver cirrhosis and/or portal hypertension.^3^ GAVE is also observed in patients after BMT and with certain cardiac diseases.^6^

Endoscopic intervention is crucial for addressing active GI bleeding in patients with GAVE. Medical management has not been shown to be an effective treatment modality, but Octreotide has been used in certain scenarios for controlling bleeding, including when endoscopic intervention is contraindicated or as a bridge to endoscopic intervention.^7^ Octreotide has been useful in our first case, temporizing further bleeding before and after endoscopic treatment. Other medications like tranexamic acid and estrogen have been tried but were not effective.^8^ Surgical options such as gastric antrectomy are typically reserved for cases where endoscopic therapy has failed.^9^

APC is the most studied and generally preferred as first‐line treatment for GAVE due to its safety and availability compared to other modalities. APC utilizes ionized gas and an electrical current to provide photocoagulation. Studies demonstrate that APC is effective in managing bleeding. Fortuna et al. in their 2022 literature review found that in 12 different studies, 90% of patients treated with APC for GAVE experienced successful outcomes.^10^ However, recurrence of bleeding in GAVE is common, and multiple treatments with APC are often required for optimal results, as seen in our first case. The number is sessions reported is variable, mostly ranging from 2 to 6 sessions. One study showed that the median number of sessions required to achieve GAVE eradications was 3.^6^ It is considered a safe method of hemostasis, as it applies superficial coagulation energy to the desired tissue. Complications such as bleeding or stricture formation are rare.

Although uncommon, GAVE is likely underrecognized in the pediatric population. Possible contributing factors include its lower incidence compared with adults, limited provider familiarity with the condition, and the potential for misdiagnosis as PHG. Further research and increased reporting are needed to enhance awareness and improve understanding of GAVE in children.

## 5 CONCLUSION

GAVE is a known cause of upper GI bleeding in adults but is comparably rare in children with few published case reports. GAVE has been associated with HSCT with few identified pediatric cases.^6^ Our case reports, including one patient with a history of HSCT, will add to existing literature and will provide additional information about GAVE in the pediatric population. It is important to consider GAVE in the differential diagnosis for GI bleeding, especially in patients with coexisting conditions such as portal hypertension or those who have undergone HSCT. Accurate differentiation of GAVE from PHG is crucial for determining the appropriate treatment approach. APC is first‐line treatment for GAVE, and in some cases, multiple treatment modalities may be necessary to achieve effective control of bleeding.

## CONFLICT OF INTEREST STATEMENT

The authors declare no conflict of interest.

## ETHICS STATEMENT

An informed consent for the publication of this case report, including any accompanying images, was obtained from the patients and/or parents.

## References

[1] Romano C , Oliva S , Martellossi S , et al. Pediatric gastrointestinal bleeding: perspectives from the Italian Society of Pediatric Gastroenterology. World J Gastroenterol. 2017;23(8):1328‐1337. 10.3748/wjg.v23.i8.1328 28293079 PMC5330817

[2] Hsu WH , Wang YK , Hsieh MS , et al. Insights into the management of gastric antral vascular ectasia (watermelon stomach). Therap Adv Gastroenterol. 2018;11:1756283X17747471. 10.1177/1756283X17747471 PMC5788127 29399041

[3] Gostout CJ , Viggiano TR , Ahlquist DA , Wang KK , Larson MV , Balm R . The clinical and endoscopic spectrum of the watermelon stomach. J Clin Gastroenterol. 1992;15(3):256‐263. 1479175 10.1097/00004836-199210000-00019

[4] Sugishita Y , Yamamoto S , Kaneko R , et al. Gastric antral vascular ectasia in a pediatric patient with neuroblastoma who underwent tandem stem cell transplantation. Blood Cell Ther. 2019;2(1):9‐11. 10.31547/bct-2018-007 37969694 PMC10645482

[5] Dulai GS , Jensen DM , Kovacs TO , Gralnek IM , Jutabha R . Endoscopic treatment outcomes in watermelon stomach patients with and without portal hypertension. Endoscopy. 2004;36(1):68‐72. 10.1055/s-2004-814112 14722858

[6] Fuccio L . Diagnosis and management of gastric antral vascular ectasia. World J Gastrointest Endosc. 2013;5(1):6‐13. 10.4253/wjge.v5.i1.6 23330048 PMC3547119

[7] Nardone G , Rocco A , Balzano T , Budillon G . The efficacy of octreotide therapy in chronic bleeding due to vascular abnormalities of the gastrointestinal tract. Aliment Pharmacol Ther. 1999;13(11):1429‐1436. 10.1046/j.1365-2036.1999.00647.x 10571598

[8] van Cutsem E , Rutgeerts P , Vantrappen G . Treatment of bleeding gastrointestinal vascular malformations with oestrogen‐progesterone. Lancet. 1990;335:953‐955. 10.1016/0140-6736(90)91010-8 1970032

[9] Belle JM , Feiler MJ , Pappas TN . Laparoscopic surgical treatment for refractory gastric antral vascular ectasia: a case report and review. Surg Laparosc Endosc Percutan Tech. 2009;19(5):e189‐e193. 10.1097/SLE.0b013e3181bb5a19 19851250

[10] Fortuna L , Bottari A , Bisogni D , et al. Gastric antral vascular ectasia (GAVE) a case report, review of the literature and update of techniques. Int J Surg Case Rep. 2022;98:107474. 10.1016/j.ijscr.2022.107474 35963152 PMC9386635

